# Changes in hydration status of elite Olympic class sailors in different climates and the effects of different fluid replacement beverages

**DOI:** 10.1186/1550-2783-10-11

**Published:** 2013-02-21

**Authors:** Evan JH Lewis, Sarah J Fraser, Scott G Thomas, Greg D Wells

**Affiliations:** 1 Department of Nutritional Sciences, University of Toronto, 150 College Street, Toronto, ON M5S 3E2, Canada; 2 Faculty of Life Sciences, McMaster University, 1280 Main Street West, Hamilton, ON L8S 4L8, Canada; 3 Faculty of Kinesiology and Physical Education, The University of Toronto, 55 Harbord Street, Toronto, ON M5R 2W6, Canada; 4 Physiology and Experimental Medicine, The Hospital for Sick Children, 555 University Avenue, Toronto, ON M5G 1X8, Canada

**Keywords:** Hydration, Sailing, Nutrition, Performance, Temperature

## Abstract

**Background:**

Olympic class sailing poses physiological challenges similar to other endurance sports such as cycling or running, with sport specific challenges of limited access to nutrition and hydration during competition. As changes in hydration status can impair sports performance, examining fluid consumption patterns and fluid/electrolyte requirements of Olympic class sailors is necessary to develop specific recommendations for these elite athletes. The purpose of this study was to examine if Olympic class sailors could maintain hydration status with self-regulated fluid consumption in cold conditions and the effect of fixed fluid intake on hydration status in warm conditions.

**Methods:**

In our cold condition study (CCS), 11 elite Olympic class sailors were provided *ad libitum* access to three different drinks. Crystal Light (control, C); Gatorade (experimental control, G); and customized sailing-specific Infinit (experimental, IN) (1.0:0.22 CHO:PRO), were provided on three separate training days in cold 7.1°C [4.2 – 11.3]. Our warm condition study (WCS) examined the effect of fixed fluid intake (11.5 mL^.^kg^.-1.^h^-1^) of C, G and heat-specific experimental Infinit (INW)(1.0:0.074 CHO:PRO) on the hydration status of eight elite Olympic Laser class sailors in 19.5°C [17.0 - 23.3]. Both studies used a completely random design.

**Results:**

In CCS, participants consumed 802 ± 91, 924 ± 137 and 707 ± 152 mL of fluid in each group respectively. This did not change urine specific gravity, but did lead to a main effect for time for body mass (p < 0.001), blood sodium, potassium and chloride with all groups lower post-training (p < 0.05). In WCS, fixed fluid intake increased participant’s body mass post-training in all groups (p < 0.01) and decreased urine specific gravity post-training (p < 0.01). There was a main effect for time for blood sodium, potassium and chloride concentration, with lower values observed post-training (p < 0.05). C blood sodium concentrations were lower than the INW group post-training (p = 0.031) with a trend towards significance in the G group (p = 0.069).

**Conclusion:**

*Ad libitum* fluid consumption in cold conditions was insufficient in preventing a decrease in body mass and blood electrolyte concentration post-training. However, when a fixed volume of 11.5 mL^.^kg^.-1.^h^-1^ was consumed during warm condition training, hydration status was maintained by preventing changes in body mass and urine specific gravity.

## Background

The maintenance of hydration status during training and competition has been repeatedly identified as a rate-limiting factor for athletic performance [1-3]. The continued intake of fluids fortified with carbohydrates and electrolytes during activities lasting longer than one hour has been found to prevent deteriorations in endurance, strength, blood volume [4-6] and cognitive function [7].

As such, the study of hydration requirements of Olympic class sailors is lacking when compared to other endurance sports such as cycling and running [8,9]. While population size and sport specific challenges may be an influencing factor, the physiologic demands of Olympic class sailing, coupled with the strategic/tactical requirements make hydration a logical variable for success that has not been adequately studied [8]. When 28 elite Olympic class sailors from New Zealand were surveyed about their sport sciences practices, 68% reported being dehydrated during racing from inadequate fluid intake that was likely related to 86% of athletes reporting a loss of concentration at the end of races and 50% reporting feelings of frustration about race results [10]. Examination of the hydration practices of novice Laser class (Men’s singlehanded Olympic dinghy) sailors competing in hot climates and moderate wind velocities, revealed participants did not consume sufficient fluids to prevent a >2% loss of body mass after racing [9], a level that has previously been associated with reduced athletic performance [3]. In both studies, the authors attributed a lack of sport science knowledge to the reported change in hydration status. Since the findings of Slater and Tan [9], we are not aware of any additional findings on the impact of environmental conditions on the hydration practices or requirements of elite or novice Olympic class sailors.

Examination of the energy demands of Laser class sailors, revealed there is a direct correlation between wind velocity and the energy demand during sailing [11]. The Laser and other Olympic class dinghies require sailors to have well-developed strength endurance, especially in the quadriceps, abdominal and upper back muscles. To navigate the boat upwind, the sailor must leverage his body out of the boat to counteract the force of the wind on the sail (for a detailed figure and description see Castagna & Brisswalter [11]). Previous examination of the physiologic response to sailing in moderate winds has determined energy requirements are largely met by aerobic metabolism. Higher skilled Laser sailors sail at 45 to 68% of maximal aerobic power during 30 or more minutes of upwind sailing in moderate conditions (14–22 km^.^h^-1^) [11,12]. Sweating rates at similar intensities measured in America’s Cup sailors can results in mean water losses of 1340 mL^.^h^-1^[13]. As there are many differences between America’s Cup and Olympic class sailing [8,13] it is important to determine the changes in hydration status and subsequent hydration requirements of Olympic class sailors.

Sweat rate and water loss are affected by environmental conditions [6] but it is unclear how sweat losses are compensated for by sailors in cold conditions. Furthermore, increased sweat losses in warm and hot conditions are not appropriately compensated for by increased fluid intake in elite football players [14,15] amateur Laser sailors [9] and America’s Cup sailors [13]. As such, the purpose of the CCS was to examine if Olympic class sailors could self-regulate fluid requirements in cold conditions by providing them *ad libitum* access to different fluid replacement beverages during training and examining how this affected hydration status. The purpose of the WCS was to test the effect of fixed fluid intake of different fluid replacement beverages on hydration status during training in warm conditions. Examining relative fluid intakes may be a novel way of developing hydration recommendations for sailors. Previous work examining the effect relative fluid intake rates on gastric emptying during cycle exercise determined that consuming 11.5 mL^.^kg^-1.^h^-1^ of a 7.5% carbohydrate solution had a higher percentage gastric emptying compared to 17.1 and 23.0 mL^.^kg^-1.^h^-1^[16]. While absolute gastric emptying in this study was greater in the higher fluid intake groups, these intakes equated to approximately 1200 and 1600 mL^.^h^-1^ and resulted in gastric discomfort [16]. Therefore, the a second purpose of this study was to determine the optimal composition of a fluid replacement drink specific to elite Olympic class sailors and test if consuming 11.5 mL^.^kg^-1.^h^-1^ was sufficient to maintain hydration status.

## Methods

### Research design

Two studies were performed to examine the changes in hydration status of elite Olympic class sailors during training. The first was a cold condition study (CCS) that examined *ad libitum* fluid consumption of three different fluid replacement drinks (Table 1) on hydration status and blood electrolyte concentration before and after training in cold (4.2 – 11.3°C) temperatures. WCS examined the effect of fixed volume (11.5 mL^.^kg^.-1.^h^-1^) fluid consumption of three different fluid replacement drinks on hydration status and blood electrolyte concentration before and after in warm temperatures (17.0 - 23.3°C). Both studies used a single blinded, placebo-controlled, repeated-measures design. Participants were randomly assigned to each condition on three separate days using a completely randomized design. In CCS*,* participants completed all three conditions over five days with a maximum of one day between conditions. Training sessions were limited to 2.5 hours because of the cold temperatures. In WCS participants completed each condition on consecutive days. All training sessions were three hours in length. In both studies, all training activities were performed in identical order for the same duration each day.

**Table 1:** Composition of experimental drinks in CCS and WCS

**Drink**	**CHO (g**^**.**^**L**^**-1**^**)**	**Protein (g**^**.**^**L**^**-1**^**)**	**CHO : PRO**	**[Na**^**+**^**] mmol**^**.**^**L**^**-1**^	**[K**^**+**^**] mmol**^**.**^**L**^**-1**^	**Energy (kcal**^**.**^**L**^**-1**^**)**
Crystal Light (C)	0	0	-	0	0	0
Gatorade (G) Study 1	66.0 [13.0 – 43.2]	0	-	18.3	3.3	264
Gatorade (G) Study 2	66.0 [59.1- 64.2]	0	-	18.3	3.3	264
Infinit (IN) Study 1	60 [6.3 – 39.3]	13.3 [3.5 - 8.7]	1.0 : 0.22	21.8	4.3	296.7
Infinit (INW) Study 2	90.0 [80.5 – 87.6]	6.7 [6.0 – 6.5]	1.0 : 0.074	72.5	21.3	386.7

Carbohydrate (CHO) and protein (PRO) content is shown with the CHO:PRO, range of ingestion per hour based on fluid consumption (Study 1) the weight of subjects (Study 2).

### Experimental drinks

CCS and WCS had three different drink conditions, Crystal Light (C) (Kraft Foods Canada, Toronto, Ontario), Gatorade (G) (Gatorade, Barrington, Illinois) and Infinit (IN) (Infinit Nutrition Canada, Windsor, Ontario). All drinks were flavoured similarly in attempts to blind the participants. The composition of the C and G conditions were consistent between both studies; however the Infinit condition was altered to reflect the hypothesized fluid replacement and electrolyte requirements of the participants determined during sweat rate testing (INW) (Table 1).

The carbohydrate content in the G drink was entirely sucrose. In the CCS, the carbohydrate content in the IN drink was approximately 60 : 40 ratio of dextrose and maltodextrin with a carbohydrate concentration of 60 g^.^L^-1^. The INW drink in WCS had a carbohydrate ratio of 2 : 1 dextrose and fructose. Protein in both drinks was whey protein isolate with 13.3 g^.^L^-1^ and 6.7 g^.^L^-1^ in the IN and INW drinks respectively.

Participants in CCS were provided *ad libitum* access to their drink condition. To measure the amount of fluid consumed during training, the content of each subject’s water bottles was measured to the nearest 1.0 mL before and after training and the difference was recorded.

In WCS participants were instructed to consume one water bottle per hour containing 11.5 mL^.^kg^.-1.^h^-1^ of fluid based on pre-training body weight. At the beginning of each hour, participants were provided with an individually pre-measured sport bottle with their respective drink and instructed to ingest all of the fluid within the hour. Each participant had a secure bottle holder in their boat to provide convenient access to their drink throughout each hour of training. Participants in both studies were asked to refrain from consuming any other fluids or food within the hour before training, during training and until all post-training measures had been completed.

### Subjects

CCS Eleven males (mean [range]) **(**age 23.3 y [19.5 – 31.6]; height 182.8 cm [177.5 - 187.0]; mass 81.5 kg [74.2 – 95.9]) were recruited for this study. All participants competed in Olympic class boats (Men’s Laser n = 6; 49er skiff n = 3; Men’s Finn n = 1 and Men’s RS:X n = 1). WCS had eight male participants that competed in the Men’s Laser (age 22.9 y [19.9 – 27.0]; height 183.4 cm [180.2 – 190.0]; mass 81.1 kg [78.8 - 84.5]). All participants in both studies had a minimum of four years experience competing at the international level in their respective class. The subjects were studied during training camps designed to replicate competitive conditions with the environmental condition being the variable between each study. Potential risks from participating in each study were explained to the subjects prior to obtaining written consent. The University of Toronto Research Ethics Board approved all study procedures.

### Sweat rate

Prior to the each study, sweat rate and sodium loss were determined during cycle exercise in controlled laboratory conditions (CCS 21.3°C, 57.4% relative humidity; WCS 21.8°C, 59.1% relative humidity). For the day of testing, participants were instructed to drink 500 mL of water upon waking, refrain from eating breakfast and report to the laboratory at 08:30. After voiding, participants were weighed to the nearest 0.1 kg (Precision Scale UC-321PL, A&D Medical, San Jose, California, USA) wearing only dry lightweight shorts. Participants had four adhesive sweat patches (Tegaderm, 3 M, London, Ontario, Canada) affixed to their, chest, upper-back, forearm and thigh to measure whole-body sodium as previously described [17]. Participants were fitted to an electronically braked ergometer (Velotron Dynafit Pro, Seattle, WA, USA) with Computrainer Software, which allowed them to adjust their resistance to maintain desired heart rate. Subjects were instructed to warm up for five minutes before completing 30 minutes of cycling. Intensity was set at 80% of age-predicted maximum heart rate (Equation 1) as this is an average heart rate observed during racing in windy conditions [18]. Patches were removed once saturated or at the conclusion of the test and sweat concentration from all patches were analyzed (Sweat Chek 3120, Wescor Biomedical Systems, Logan, Utah, USA). This protocol produced profuse sweating in all participants and was similar to previously validated testing procedures [19].

### Blood electrolytes

In CCS finger prick blood samples were collected into heparinized capillary tubes for immediate analysis in CHEM8+ cartridges inserted into an iSTAT point of care monitor (Abbott, Princeton, NJ, USA). The CHEM8+ cartridge analyses sodium, potassium, chloride, glucose, hematocrit and hemoglobin as previously described [20]. In WCS*,* venous blood samples were collected from the antecubital vein into heparinized tubes. Two participants were uncomfortable with venous sampling so finger prick blood samples were collected as described in CCS. Samples were pipetted into iSTAT CHEM8+ cartridges and analyzed as described in CCS.

### Hydration status

Before and after training, participants provided a midstream urine sample in a polyurethane collection container for immediate analysis of urine specific gravity (USG) in triplicate (4410 PAL-10S, Novatech International, Houston, Texas, USA). At this time, participants voided completely and were then weighted to the nearest 0.1 kg (Precision Scale UC-321PL, A&D Medical, San Jose, California, USA), wearing only dry lightweight shorts. Differences in body mass were used to estimate hydration status and to calculate sweat rate (Equation 3). Urine excreted by each participant during training in WCS was collected in a large airtight container, carried by a support boat. There was no correction made for respiratory water loss or metabolic fluid changes. Changes in plasma volume were calculated using changes in hematocrit and hemoglobin according to the methods of Dill and Costill [21].

### Environmental conditions

Environmental conditions were measured every 30 minutes during training using a portable weather station with anemometer (Kestrel 4000, Nielsen-Kellerman, Mckellar, Australia).

### Calculations

Participant’s target heart rate during sweat rate testing was calculated by subtracting participants’ age from 220 and then multiplying by 80%.

(1) $$\mathrm{Heart}\;\mathrm{rate}=\left(220-\mathrm{age}\right)*0.80$$

Mean whole body sodium output was calculated based on the equation of Patterson et al. [17].

(2) $$\begin{array}{l} \mathrm{Whole}-\mathrm{body}\left[\mathrm{Na}\right]=28.2\%\;\mathrm{chest}\left[\mathrm{Na}\right]\\ \;+28.2\%\;\mathrm{back}\left[\mathrm{Na}\right]\\ \;+11.3\%\;\mathrm{forearm}\left[\mathrm{Na}\right]\\ \;+32.3\%\;\mathrm{thigh}\left[\mathrm{Na}\right] \end{array}$$

This data was pooled and used as a guide to determine the electrolyte content of the Ex drink (Table 1).

Sweat rate (millilitres per hour) was estimated as change in body mass (kilograms), with the assumption 1 kg = 1 L, during the 3 hour practice plus total fluid intake (milliliters) and minus total urine output during practice (millilitres).

(3) $$\begin{array}{l} \mathrm{Sweat}\;\text{rate}=\left(\mathrm{Pre}\;\mathrm{body}\;\mathrm{mass}-\mathrm{Post}\;\mathrm{body}\;\mathrm{mass}\right)\\ \;\frac{+\mathrm{Fluid}\;\mathrm{intake}-\mathrm{Urine}\;\mathrm{output}}{\mathrm{Practice}\;\mathrm{length}} \end{array}$$

Total sweat sodium loss (grams) for participants was calculated by multiplying their sweat sodium concentration (millimoles per litre) with the molecular weight of sodium (22.99 grams per mol) with the total sweat volume lost (litres).

(4) $$\begin{array}{l} \mathrm{Sodium}\;\mathrm{loss}=\mathrm{Sweat}\;\mathrm{sodium}\;\mathrm{concentration}*22.99\\ \;*\mathrm{Sweat}\;\mathrm{loss} \end{array}$$

The total sodium intake (grams) of each participant was calculated by multiplying the sodium concentration of each drink (Table 1) with the molecular weight of sodium (22.99 grams per mol) with the total volume of each drink consumed (litres).

(5) $$\begin{array}{l} \mathrm{Sodium}\;\mathrm{intake}=\mathrm{Drink}\;\mathrm{sodium}\;\mathrm{concentration}\\ \;*22.99*\mathrm{Fluid}\;\mathrm{intake} \end{array}$$

### Statistical analysis

Data is presented as the mean [range] for all descriptive statistics and mean ± SE for comparison between and within conditions with the level of confidence set at p < 0.05 to determine significance. Differences from pre to post training between and within conditions were examined first using a multivariate analysis of variance (MANOVA) for the blood electrolytes and hemoglobin concentrations. Analysis of variance (ANOVA) tests were then applied to each variable to determine which conditions differed and a Tukey’s honestly significant difference post-hoc analysis applied when appropriate. Electrolytes with no differences detected using MANOVA, blood glucose, USG and body mass changes were analyzed using repeated measures ANOVA. There was no difference between the athletes sailing different boats in CCS so all participants were pooled into a single group. In WCS, participants’ sweat rate and sodium balance variables and glucose intake were analyzed using a one-way ANOVA with Tukey’s honestly significant difference. Analysis was performed using SPSS version 20.

## Results

### Cold condition study

#### Environmental conditions

During training the wet bulb temperature was 7.1°C [4.2 – 11.3] with 62.7% [32 – 87] relative humidity. Wind velocity was 23.5 km^.^h^-1^ [17.0 - 36.9].

#### Hydration status

Pre-training USG values showed that participants arrived for training in a borderline hypohydrated state. There were at least three participants in each group that had USG values greater than 1.025. Examination of USG after training showed no effect of time (p = 0.318) (Table 2). At least two participants per group had USG values greater than 1.025. Measurement of plasma volume supports our USG measurements, as there was no difference from pre- to post-training (p = 0.871). Participants consumed an average of 811.1 mL [242–1638] of fluid during training (Table 2). This resulted in an average decrease in body mass of 0.40 kg [0 – 1.0]. Body mass changes were not different between groups but there was a main effect for time (p < 0.001).

**Table 2:** Changes hydration status measured during the CCS

	**Crystal Light (C)**	**Gatorade (G)**	**Infinit (IN)**
USG pre (AU)	1.021 ± 0.002	1.019 ± 0.003	1.020 ± 0.003
USG post (AU)	1.018 ± 0.003	1.019 ± 0.002	1.020 ± 0.002
Fluid Intake (mL)	802 ± 91 [242 – 1110]	924 ± 137 [493 – 1638]	707 ± 152 [186 – 1638]
Change in plasma volume (%)	3.2 ± 2.4	5.4 ± 2.7	4.8 ± 6.7
Change in body mass (kg) *	−0.5 ± 0.1 [0 – -1.0]	−0.4 ± 0.1 [−0.2 – -0.1]	−0.4 ± 0.1 [0 – -0.7]

*Main effect for time. Significantly different from pre-sailing values (p < 0.001).

Data is presented as mean ± SEM [range].

#### Hematological measurements

**Figure 1:**
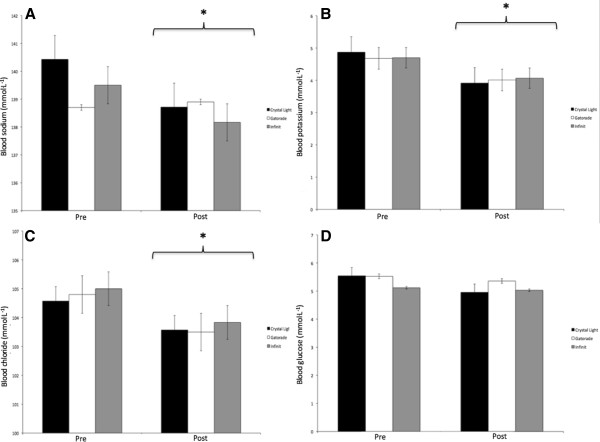
**Changes in blood variables from the cold condition study (CCS). A** – Blood sodium concentration, **B** – Blood potassium concentration, **C** - blood chloride concentration, **D** – Blood glucose concentration. * Above a bracket indicates a main effect for time (p < 0.05). All data are shown as mean ± SE.

Blood sodium concentrations were lower post-training with a main effect for time (p = 0.02). The group by time interaction for sodium trended toward significance (p = 0.084) (Figure 1A). Participants’ blood potassium concentration were lower after training C −19.4%, G −13.7% and IN −13.0%, with a main effect for time (p < 0.001) (Figure 1B) and blood chloride concentrations also lower after training with a main effect for time (p = 0.007) (Figure 1C). There was a trend towards a main effect for time for blood glucose (p = 0.074) (Figure 1D).

### Warm condition study

#### Environmental conditions

Wet bulb temperature during training was 19.5°C [17.0 - 23.3] with 82.3% relative humidity [76.0 – 89.1]. Wind velocity was 24.3 km^.^h^-1^ [15.0 – 36.0).

#### Hydration status

Examination of USG and body mass revealed a main effect for time for both measures respectively (p = 0.01, p = 0.003) with no difference between drink conditions (Table 3). Before training participants’ USG was higher in all groups with the C and G groups having near hypohydrated values of 1.019 and 1.020 respectively. Two participants in the C group had USG values >1.030. Body mass increased after training for all groups (p = 0.01). Participants gained an average of 0.31 kg or 0.44% body mass.

**Table 3:** Changes of hydration variables measured in the WCS

	**Crystal Light (C)**	**Gatorade (G)**	**Infinit (INW)**
USG pre (AU)	1.020 ± 0.003	1.020 ± 0.002	1.018 ± 0.002
USG post (AU)^c^	1.015 ± 0.006	1.007 ± 0.002	1.014 ± 0.002
Change in body mass (kg)	0.3 ± 0.1	0.4 ± 0.2	0.3 ± 0.2
Change in hemoglobin (%)^+^	−4.1 ± 1.5	−7.5 ± 1.6	−4.5 ± 3.0^b^
Change in plasma volume (%)^+^	1.3 ± 0.28	1.7 ± 0.33	1.5 ± 0.82
Sweat rate (mL^.^h^-1^)	510.1 [20.9 -841.1]	597.3 [401.1 – 848.0]	727.2 [456.2-849.0]
Sodium intake (g)*	0	1.2 [1.1 – 1.2]	4.7 [4.4 – 4.7]
Sodium loss (g)	3.1 [0.94 – 5.9]	3.7 [2.0 – 5.8]	4.9 [2.0 – 7.4]
Sodium balance (g)	−3.1 [−4.4 – 0.94]	−2.5 [2.9 - -0.77]	−0.23 [−1.2 – 2.7]^a^

* - All groups are significantly different from each other (p < 0.001). a – Significantly different from Crystal Light (p = 0.022). + Main effect for time indicating that there was an increase in plasma volume in all groups following training (p < 0.001). b – Significantly different from pre-training (p < 0.05). c – different from pre-training USG with a main effect for time (p = 0.003).

Values are shown as the mean (range) for each condition.

Blood hemoglobin concentration was significantly lower in the G group after training when compared to controls pre-training (p > 0.05) (Table 3). When changes in hemoglobin and hematocrit were used to calculate changes in plasma volume there was a main effect for time (p < 0.001), indicating a significant increase from pre to post-training; however, there were no differences between groups (Table 2).

#### Electrolytes

**Figure 2:**
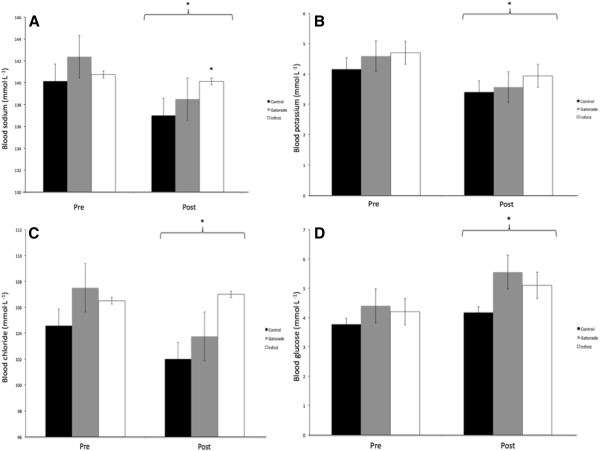
**Change in blood variables for the warm condition study (WCS). A** - Blood sodium concentration (* significantly different from Control p = 0.031), **B** - Blood potassium concentration, **C** - Blood chloride concentration and **D** - Blood glucose. * Above a bracket indicates a main effect for time (p < 0.05). All data are shown as mean ± SE.

Blood sodium concentrations were reduced 2.6% in the C and 2.3% in the G condition when compared to INW (p = 0.031, p = 0.069) (Figure 2). Post-training sodium concentration was different between C and INW conditions only (p = 0.031) (Figure 2A). Sodium intake was different between each group; however, the amount lost through sweat was not different (Table 2). This resulted in only the INW group having a near neutral sodium balance compared to C and G groups (p = 0.022) There was a main effect for time for both blood potassium and chloride concentration (p < 0.001, p < 0.001) (Figure 2). One-way ANOVA of the post-training measurements of these electrolytes suggested a trend towards difference in groups for chloride (p = 0.072).

#### Blood glucose

Despite the different carbohydrate concentrations between groups, there was no difference between conditions for blood glucose levels (Figure 2D). A main effect for time was found (p = 0.006), suggesting an increase in blood glucose after training.

## Discussion

The present studies measured changes in hydration status of elite Olympic class sailors in cold and warm conditions. CCS revealed participants consumed insufficient fluids to prevent a decrease in body mass during training, regardless of drink condition, causing a reduction in blood electrolyte concentration. WCS showed that consuming 11.5 mL^.^kg^-1.^h^-1^ of fluid from any condition prevented a decrease in body mass, lowered USG in all conditions and blood sodium concentration and sodium balance were maintained with the custom drink condition (INW) only.

### Hydration

The average pre-training USG value for all groups in both studies was 1.019 (Table 2 and 3), which is very close to the 1.020 threshold that has been associated with hypohydration [22]. As participants were encouraged to consume fluids ad libitum prior to training, this finding suggests individual practices are inadequate. Hamouti et al. [23] have suggested an athlete’s muscle mass may influence USG values and therefore a USG measurement of 1.020 may not be an accurate cut-off for hypohydration. While developing an exact cut-off for hypohydration in athletes given their developed muscle mass compared the average population may require further study, the observed pre-practice USG values recorded during both studies were at the higher end of optimal. Since training began at 11:00 am daily, there was adequate time for athletes to consume fluids prior to arriving at the sailing centre. Furthermore, the variability between participants in pre-training USG measurements, especially in the WCS, favours inadequate fluid consumption as opposed to a higher rate of urine protein metabolites due to high muscle mass.

In the WCS, participants’ fluid intake was standardized to 11.5 mL^.^kg^-1.^h^-1^ to reflect previous recommendations on relative fluid intake [16] and enable the comparison of hydration status and sodium balance between subjects and drinks. The decision to standardize participants’ fluid intake was also based partially on the variability of fluid intake observed during the CCS and from inadequate fluid intake reported in previous studies [9,14]. A leading cause of insufficient fluid intake for athletes training and competing in cold temperatures is reduced thirst, which is restored in warm conditions [24]. Examination of elite football players training in cool (5°C) temperatures revealed athletes consumed far less fluid than was lost from sweating [15]. Furthermore, runners have been found to underestimate their sweat rates in cool conditions, despite accurate estimations of their fluid consumption [25]. These and our findings suggest athlete’s perception of sweat rates in cool climates is impaired, which reinforces the need for specific hydration guidelines. The fluid requirements of participants in WCS (19.5°C [17.0 - 23.3]), were anticipated to reflect the average laboratory sweat rate of 1470 mL^.^h^-1^ measured at 21.8°C. The fluid intake rate of 11.5 mL^.^kg^-1.^h^-1^ was selected to deliver approximately 65% of the average laboratory sweat rate and a volume less than one litre (906.2 – 971.8 mL^.^h^-1^), with a carbohydrate content between 6-9%. This range of carbohydrate consumption in fluid replacement drinks has been identified as an optimal range for absorption and gastric emptying [6]. Furthermore, consuming volumes greater than 1000 mL^.^h^-1^ during exercise has caused gastro-intestinal discomfort in highly trained individuals [26]. None of the participants in the study commented on any bloating or gastro-intestinal issues during or after training.

Surprisingly, participants’ average on-water sweat rate was only 611.8 ± 47.2 mL^.^h^-1^. This was 41.5% lower than the pre-study laboratory sweat rate of 1470 mL^.^h^-1^. As a result, participants mean fluid intake was 933.33 ± 5.13 mL^.^h^-1^ or 153.0% fluid replacement. Since on-water temperatures were similar to that of the laboratory sweat rate testing, it appears the cooling effect of splashing waves and brief pauses in activity between training drills did not elicit the same physiologic sweat response during sailing as seen during cycle exercise. This suggests laboratory based sweat rate testing over estimates sweat rates observed on-water in this study. Therefore, the on water environmental conditions experienced by Olympic class sailors may have a direct modulating influence on sweat rate and fluid requirements. Based on our observations, a lower fluid replacement rate would be more appropriate for the conditions experienced in this study. Extrapolating from the data presented, a fluid intake rate of 7.4 mL^.^kg^-1.^h^-1^ would achieve the desired hydration state.

### USG and electrolytes

The greater fluid consumption compared to fluid loss during WCS may account for some of our results. Analysis of USG showed an effect for time (p = 0.003) with lower values after training in all groups (Table 3). This was coupled with a main effect for time for body weight, whereby all groups increased body mass during training as direct result of fluid intake. This was a clear difference from CCS during which there was no difference in USG and a decrease in body mass post-training (p < 0.001). In CCS it was not surprising to see no difference between groups for measures of hydration status; however, given the 3 and 4 fold higher concentrations of sodium and potassium between the INW and G drink conditions in WCS, we anticipated a difference between groups post-training. While it is clear that all groups retained fluid during training, an effect of the positive fluid balance, the higher sodium concentration in the INW condition did not have a compounding effect on fluid retention as could have been anticipated as observed in previous findings [27].

The sweat sodium loss of participants in WCS (Table 3) is similar to values reported by other groups studying elite athletes [15,28]. While there was no difference in sodium loss with the different drinks, sodium balance was almost unchanged in the INW group compared to C and G conditions. This was a result of the INW drink being designed for full sodium replacement. Sodium intake is essential for the absorption and retention of fluid during exercise [27]. Results from hydration testing in other sports have shown elite athletes have difficulty replacing sodium lost during training using fluid replacement drinks [19,29]. These finding, coupled with our results from CCS, can be explained in part by the ad libitum fluid consumption study protocol. This indicates athletes may have difficulty self-regulating their hydration requirements particularly in cold conditions, as it is easy to become caught-up in the focus and intensity of training and/or competition. This further supports the need for individual, sport specific or relative fixed volume fluid replacement recommendations.

### Blood glucose carbohydrates intake

Examination of the energy demands of Laser sailing by Castagna and Brisswalter [11] revealed aerobic metabolism is the main energy source used by elite sailors to fulfill muscle energy demands. As such, blood glucose levels in CCS were trending towards a decrease over time (p = 0.074), despite the supply of exogenous carbohydrates in the G and IN groups; although, the average carbohydrate intake in these groups was only 61 g and 42 g respectively. Interestingly, the blood glucose concentration of the C group was stable through the 2.5 h training session despite consuming no exogenous carbohydrates (Figure 1D). In comparison, trained cyclists working at 74% VO_2_max in laboratory conditions experienced a significant decrease in blood glucose after 90 minutes of cycling [30]. Examination of substrate metabolism during 60 minutes of cycling at 70% VO_2_max at 0°C revealed almost 60% of energy expenditure was from carbohydrate metabolism [31]. This level was maintained regardless of infused non-esterified fatty acids, suggesting that carbohydrates are a preferred source of energy in cold conditions as fatty acid metabolism has been found to increase based on substrate availability in temperature environments [32]. While the intensity of Laser sailing in conditions similar to CCS reached approximately 65% VO_2_max [11], this difference in intensity may have been enough to prevent deleterious changes in blood glucose in the C condition.

In WCS, blood glucose levels were surprisingly unchanged between the drink conditions (Figure 2D). Although a main effect for time was observed (p = 0.006), indicating that blood glucose was higher after training, this was largely driven by the G and INW groups. The carbohydrate content in the G drink was 66 g^.^L^-1^, which is approximately in-line with the current American College of Sports Medicine recommendations [4]. These guidelines were based on the understanding that carbohydrates ingested during exercise could only be oxidized at a maximum rate of 1 g^.^min^-1^[33]. However, advances in carbohydrate metabolism research have determined up to 1.75 g^.^min^-1^ can be oxidized when using multiple transportable carbohydrates, such as glucose and fructose [34]. As such, the carbohydrate content in the INW drink was comprised of glucose and fructose delivered in a 2:1 ratio at 1.3 – 1.5 g^.^min^-1^ based on a concentration of 90 g^.^L^-1^. Previous work has determined this ratio of carbohydrate delivered in solution and ingestion at 1.5 g^.^min^-1^ can improve exogenous carbohydrate metabolism during exercise by 13% [35] to 48% [36] compared to consuming an isocaloric glucose only solution. While carbohydrate oxidation was not measured in this study, consuming a drink with high carbohydrate concentration using multiple transporters has a potentially powerful effect for sailing athletes, as World Cup regattas last 5–7 days with up to three hours of competitions per day. Therefore, reducing endogenous carbohydrate oxidation could potentially preserve stored muscle glycogen energy for later in the competition, which has previously been found to have a performance enhancing effect [37].

During competition, sailors can spend anywhere from two hours to six hours on-water, with time divided between warm-up, racing and waiting for changes in wind and weather and cool-down. Given the length of time on-water, the co-ingestion of carbohydrates and protein is necessary to prevent extended periods of muscle protein breakdown. Research examining the addition of whey protein to carbohydrate electrolyte beverages has revealed inconsistent results for improved athletic performance in both acute exercise [38,39] and cycling time trials [40,41]. In these studies, the addition of protein to an experimental beverage was focused on improving athletic performance in acute exercise. In contrast, the addition of protein to a carbohydrate electrolyte drink used during multi-day competitions may be more appropriate for metabolic reasons and worthy of continued investigation. Saunders et al. [42] found the use of a fluid replacement drink fortified with protein during a two cycle-to-exhaustion tests within the same day was effective in attenuating the nutritional deficit incurred during exercise and helped to reduce skeletal muscle damage compared to a carbohydrate electrolyte drink alone. Therefore, performing multiple bouts of exercise within a day or consecutive days of competition may be necessary to fully observe the nutritional and physiologic effects of protein ingested with a carbohydrate electrolyte beverage during exercise [43].

### Limitation

The authors acknowledge there were several limitations to the present study. The sample size for both studies was calculated to detect electrolyte changes. Based on subject variability and the applied nature of this research additional subjects would have been beneficial to detect differences between conditions; however, the maximum number of available participants was recruited.

## Conclusion

Participants in the ad libitum design CCS were unable to maintain hydration status in any condition due to inadequate fluid consumption. This may have resulted from a reduced desire to drink and/or poor estimation of individual hydration requirements in cold temperatures. When 11.5 mL^.^kg^-1.^h^-1^ of fluid was consumed in the WCS, all conditions improved urinary markers of hydration and prevented a loss of body mass. The C and G conditions were unable to maintain blood electrolyte concentrations while the customized INW condition was effective in maintaining blood sodium concentrations but not potassium. This was the first study to test relative fluid intake based on laboratory sweat rate on the hydration requirements of Olympic class sailors in warm conditions. Therefore, it is important to note that laboratory sweat testing results did not directly correspond with on-water sweat rate. This finding may guide further research of the hydration requirements of sailors in different environmental conditions.

## Competing interests

The authors declare that they have no competing interests.

## Author contributions

EJHL, SGT and GDW participated in study conception and design. EJHL and SJF performed data collection. EJHL performed statistical analysis and data analysis with SGT and GDW. All authors participated in writing, editing and approval of the final manuscript.
